# Recent advances in the management of anaplastic thyroid cancer

**DOI:** 10.1186/s13044-020-00091-w

**Published:** 2020-11-24

**Authors:** Simone De Leo, Matteo Trevisan, Laura Fugazzola

**Affiliations:** 1 Department of Endocrine and Metabolic Diseases, IRCCS Istituto Auxologico Italiano, Piazzale Brescia, 20, 20149 Milan, Italy; 2 Department of Pathophysiology and Transplantation, Università degli Studi di Milano, Milan, Italy

**Keywords:** Anaplastic Thyroid Cancer, Treatment, Tyrosine-kinase Inhibitors, Immunotherapy, Immune Checkpoint Inhibitors, Targeted Therapy

## Abstract

Anaplastic thyroid cancer (ATC) is undoubtedly the thyroid cancer histotype with the poorest prognosis. The conventional treatment includes surgery, radiotherapy, and conventional chemotherapy. Surgery should be as complete as possible, securing the airway and ensuring access for nutritional support; the current standard of care of radiotherapy is the intensity-modulated radiation therapy; chemotherapy includes the use of doxorubicin or taxanes (paclitaxel or docetaxel) generally with platin (cisplatin or carboplatin). However, frequently, these treatments are not sufficient and a systemic treatment with kinase inhibitors is necessary. These include multitarget tyrosine kinase inhibitors (Lenvatinib, Sorafenib, Sunitinib, Vandetanib, Axitinib, Pazopanib, Pyrazolo-pyrimidine compounds), single target tyrosine kinase inhibitors (Dabrafenib plus Trametinib and Vemurafenib against BRAF, Gefitinib against EGFR, PPARγ ligands (e.g. Efatutazone), Everolimus against mTOR, vascular disruptors (e.g. Fosbretabulin), and immunotherapy (e.g. Spartalizumab and Pembrolizumab, which are anti PD-1/PD-L1 molecules). Therapy should be tailored to the patients and to the tumor genetic profile. A BRAF mutation analysis is mandatory, but a wider evaluation of tumor mutational status (e.g. by next-generation sequencing) is desirable. When a BRAF^V600E^ mutation is detected, treatment with Dabrafenib and Trametinib should be preferred: this combination has been approved by the Food and Drug Administration for the treatment of patients with locally advanced or metastatic ATC with BRAF^V600E^ mutation and with no satisfactory locoregional treatment options. Alternatively, Lenvatinib, regardless of mutational status, reported good results and was approved in Japan for treating unresectable tumors. Other single target mutation agents with fair results are Everolimus when a mutation involving the PI3K/mTOR pathway is detected, Imatinib in case of PDGF-receptors overexpression, and Spartalizumab in case of PD-L1 positive tumors. Several trials are currently evaluating the possible beneficial role of a combinatorial therapy in ATC. Since in this tumor several genetic alterations are usually found, the aim is to inhibit or disrupt several pathways: these combination strategies use therapy targeting angiogenesis, survival, proliferation, and may act against both MAPK and PI3K pathways. Investigating new treatment options is eagerly awaited since, to date, even the molecules with the best radiological results have not been able to provide a durable disease control.

## Background

Anaplastic thyroid cancer (ATC) is the rarest type of thyroid cancer but also the deadliest. Its incidence has been constant during the last four decades and it accounts for around 1–2% of all thyroid cancer diagnoses [1, 2]. Patients report the appearance, in a period of days or few weeks, of a growing neck mass, associated with dysphagia, dyspnea, hoarseness, and neck pain. Sometimes an urgent intervention is needed to prevent death by asphyxiation. ATCs are considered to derive from differentiated thyroid cancers (DTC) or poorly differentiated thyroid cancers (PDTC) and therefore may maintain the mutations of the tumor from which they derive. However, histologically, ATCs do not present any of the morphological features of follicular cells [3]. The eighth version of the American Joint Committee on Cancer (AJCC), published in October 2016, updated the AJCC/TNM cancer staging system [4]. Unlike the previous edition, where all ATCs were classified as T4 tumors, in the eight edition T definition follows the same rules of differentiated thyroid cancers. The stage for ATC is always IV and is divided in IV A when ATC is only intrathyroidal, IV B when there is a gross extrathyroidal extension or cervical lymph node metastases, IV C when there are distant metastases [4]. The ATC Research Consortium of Japan evaluated a database of more than 750 ATC patients and assessed overall survival (OS), applying the 8th edition. The IV A, IV B, and IV C group of patients had a median OS of 15.8, 6.1, and 2.8 months, respectively [5].

The management of patients with ATC should be in the hands of a multidisciplinary team, which should account Endocrinologists and/or Oncologists, Surgeons, Radiotherapists, Radiologists, and Psychologists. The best approach should be, when feasible, the complete resection of the lesion. The criteria used for determining if the tumor is resectable are based on the evaluation of both the tumor burden, and the extent of invasion of the structures involved [6]. Surgery, radiotherapy and conventional chemotherapy are commonly used in ATC [6, 7]. Because of the aggressive behavior of this tumor and the poor outcome with conventional therapies, new treatments have been tested in phase II and phase III trials (Table 1). Their efficacy has been commonly evaluated according to the Response Evaluation Criteria in Solid Tumors (RECIST) criteria, where the best morphological response of target lesions is graded in a four-class response: complete response (CR), partial response (PR), stable disease (SD), and progressive disease (PD) [8]. In general and in this paper, the objective response rate (ORR) includes patients with CR and PR, and the disease control rate (DCR) patients with CR, PR, and SD. All these new drugs have a various toxic profile (Table 2). Adverse events are classified according to the Common Terminology Criteria for Adverse Events, last version being the 5.0 [9]. Unfortunately, only a minority of these molecules have been approved in some Countries, and the opportunities to be enrolled in clinical trials are still limited, especially in Europe.

**Table 1:** Panoramic view of clinical trials and retrospective studies with novel drugs for anaplastic thyroid cancer

Pharmacodynamic Property		Tested Drug(s)	First Author	Study Design	ATC patients N° (out of total)	Prior ATC cohort therapies (%)			ATC Staging (%)			ORR (%)	DCR (%)	Median OS, months
						**CTX**	**XRT**	**Surgery**	**IVA**	**IVB**	**IVC**			
mTKI		**Lenvatinib**	Tahara	Phase II	17 (out of 51)	7 (41)	9 (53)	14 (82)	4 (24)	5 (29)	6 (35)	4/17 (24)	16/17 (94)	10.6
			Iwasaki	Retrospective	23 (out of 23)	0 (0)	0 (0)	10 (43)	0 (0)	0 (0)	23 (100)	4/23 (17)^A^	10/23 (44)^A^	5.5^B^
			Iyer	Retrospective	16 (out of 16)	9 (69)	7 (44)	8 (50)	0 (0)	4 (25)	12 (75)	3/10 (30)	7/10 (70)	3.9
		**Sorafenib**	Savvides	Phase II	20 (out of 20)	20 (100)	18 (90)	18 (90)	0 (0)	0 (0)	20 (100)	2/20 (10)	7/20 (35)	3.9
			Ito	Phase II	10 (out of 18)	6 (60)	7 (70)	7 (70)	1 (10)	0 (0)	8 (80)	0/10 (0)	4/10 (40)	5.0
		**Sunitinib**	Ravaud	Phase II	4 (out of 71)	NA	NA	NA	0 (0)	0 (0)	4 (100)	0/4 (0)	1/4 (25)	NA
		**PP-compounds** **CLM3/24/29**	**-**	**-**	**-**	**-**	**-**	**-**	**-**	**-**	**-**	**-**	**-**	**-**
		**Vandetanib**	**-**	**-**	**-**	**-**	**-**	**-**	**-**	**-**	**-**	**-**	**-**	**-**
		**Axitinib**	Cohen	Phase II	2 (out of 60)	NA	NA	NA	NA	NA	NA	1/2 (50)	1/2 (50)	NA
		**Pazopanib**	Bible	Phase II	15 (out of 15)	11 (73)	12 (80)	NA	0 (0)	1 (7)	14 (93)	0/15 (0)	0/15 (0)	3.7
		**Imatinib**	Ha	Phase II	11 (out of 11)	9 (82)		7 (64)	0 (0)	5 (45)	6 (55)	2/8 (25)^C^	6/8 (75)^C^	NA^D^
Single target	EGFR	**Gefitinib**	Pennell	Phase II	5 (out of 27)	3 (60)	NA	NA	0 (0)	5 (100)		0/5 (0)	1/5 (20)	NA
	BRAF	**Vemurafenib**	Hyman	Phase II	7 (out of 122)	7 (100)	6 (86)	NA	NA	NA	NA^E^	2/7 (28)	2/7 (28)	NA
		**Dabrafenib +** **Trametinib**	Subbiah	Phase II	16 (out of 100)	6 (38)	13 (81)	14 (88)	0 (0)	16 (100)		11/16 (69)	14/16 (88)	NA
			Iyer	Retrospective	16 (out of 16)	9 (69)	7 (44)	8 (50)	0 (0)	4 (25)	12 (75)	3/6 (50)	5/6 (83)	9.3
	mTOR	**Everolimus**	Lim	Phase II	6 (out of 40)	2 (33)			0 (0)	6 (100)		0/6 (0)	0/6 (0)	NA
			Schneider	Phase II	7 (out of 35)	NA	NA	NA	NA	NA	NA	0/7 (0)	0/7 (0)	2.8
			Hanna	Phase II	7 (out of 50)	3 (43)	4 (57)	5 (71)	0 (0)	0 (0)	7 (100)	1/7 (14)	3/7 (43)	4.6
	PPARγ	**TZDs**	**-**	**-**	**-**	**-**	**-**	**-**	**-**	**-**	**-**	**-**	**-**	**-**
	VEGFR-2	**Cyclic amide CLM94**	**-**	**-**	**-**	**-**	**-**	**-**	**-**	**-**	**-**	**-**	**-**	**-**
Vascular disrupting		**Fosbretabulin**	Mooney	Phase II	26 (out of 26)	13 (50)	17 (65)	19 (73)	7 (27)		19 (73)	0/26 (0)	7/26 (27)	4.7
Immunotherapy (anti PD-1/PD-L1)		**Spartalizumab**	Capdevila	Phase II	38 (out of 42)^F^	25 (60)	30 (71)	28 (67)	NA	NA	NA	8/42 (19)	13/42 (31)	5.9
Combination therapy		**Sorafenib +** **Temsirolimus**	Sherman	Phase II	2 (out of 36)	NA	NA	NA	NA	NA	NA	1/2 (50)	1/2 (50)	NA
		**Efatutazone + Paclitaxel**	Smallridge	Phase I	15 (out of 15)^G^	4 (27)	8 (53)	11 (73)	0 (0)	4 (27)	11 (73)	0/7 (0) vs. 1/6 (17)	4/7 (57) vs. 4/6 (67)	3.3 vs. 4.6
		**Fosbretabulin +** **CP**	Sosa	Phase II/III	80 (out of 80)	19 (35)	21 (38)	30 (55)	1 (2)	4 (7)	49 (89)	11/55 (20)	22/55 (40)	5.2

Abbreviations: *CTX* Chemotherapy (including both traditional and novel drugs); *XRT *Radiotherapy; *ORR* Overall Response Rate; *DCR* Disease Control Rate; *OS* Overall Survival; *mTKI* multiple Tirosine-Kinase Inhibitor; *PP *Pyrazolo [3,4-d]pyrimidine; *EGFR* Epidermal Growth Factor Receptor; *mTOR* mammalian Target Of Rapamycin; *PPARγ* Peroxisome Proliferator-Activated Receptor Gamma; *TZDs* Thiazolidinediones; *VEGFR* Vascular Endothelial Growth Factor Receptor; *PD-1* Programmed cell Death protein 1; *PD-L1* Programmed death-ligand 1; *CP* Paclitaxel followed by Carboplatin.

NOTES

^A^BOR was not evaluable in 6 patients

^B^Median OS in patients treated surgically vs. patients treated with Lenvatinib only was 8.8 months vs. 4.3 months, respectively

^C^Only 8 of 11 enrolled patients were evaluable for BOR

^D^The rate of 6-month OS was 45%^E^At least 6 patients (86%) presented at distance metastasis

^F^All data reported refer to the full cohort of patients enrolled, including 4 whose diagnosis of ATC was not histologically confirmed. Most patients had metastatic disease, with lungs and lymph nodes as the most common sites

^G^ORR, DCR and OS refer to Paclitaxel + Efatutazone 0.15 mg vs. Paclitaxel + Efatutazone 0.3 mg cohorts, respectively (two patients adimistered with Efatutazone 0.5 mg only incurred disease progression during the run-in phase)

**Table 2:** Toxic profile of targeted therapy tested in anaplastic thyroid cancer

Evaluated drug(s)	AEs grade I/II Recurrence (%)	AEs grade III/IV/V Recurrence (%)	REFERENCE(s)
**Lenvatinib**^**A**^	Fatigue, Hypertension (38–53), Decreased appetite (25–64), Nausea (13–59), Proteinuria (19–53), Mucositis/Stomatitis (25–41)	Hypertension (6–29), Decreased appetite (0–18), Thrombocytopenia (0–12), Fatigue (6)	Tahara et al. Iyer et al.
**Sorafenib**^**A**^	Weight decreased (50–55), Rash/desquamation (40–55), Fatigue (30–55), Anemia (0–55), Palmar-plantar erythrodysesthesia (0–50)	Hypertension, AST increase (0–20), Rash/desquamation (0–15), Hyponatremia (0–10), Hypophosphatemia (0–10)	Savvides et al. Ito et al.
**Sunitinib**^**B**^	Asthenia/fatigue (56), Mucosal AEs (53), Diarrhea (48), Cutaneous AEs (44), Hemorrhage (37)	Asthenia/fatigue (27), HFS (18), Neutropenia (17), Diarrhea (13), Mucosal AEs (11)	Ravaud et al.
**Pazopanib**^**A**^	Fatigue (73), Anorexia (53), Diarrhea (47), Hypertension, Nausea (40), Protein urine positivity, Skin Hypopigmentation (33)	Hypertension, Pharyngo-laryngeal pain (13), Alanine/asprtate aminotransferase increase, Atrial fibrillation, Leukocyte/Lymphocyte count decrease, Thrombosis (7)	Bible et al.
**Gefitinib**^**B**^	Rash (44), Diarrhea (37), Nausea (19), Anorexia (11)	Rash (7), Diarrhea (4)	Pennell et al.
**Vemurafenib**^**A**^	Rash, Decreased appetite, Dysphagia (43), Arthralgia, Candida infection, Photosensitivity reaction, Cough, Cognitive disorder, Pruritus, Vomiting, Pyrexia, Palmar-plantar erythrodysesthesia Syndrome (29)	Fatigue (29), Rash, Pyrexia, Dehydration, Cutaneous squamous cell carcinoma (14)	Hyman et al.
**Imatinib**^**A**^	Myalgia/arthralgia, Abnormal liver function test (73), Electrolyte abnormality (64), Fatigue, Anemia (55), Cough, Lymphopenia, Edema, Dyspnea (45), Nausea/Vomiting, Hyperglycemia (36)	Lymphopenia (45), Edema (27), Electrolyte abnormality, Syncope, Electrolyte abnormality, Nausea/Voimiting, Anemia (18)	Ha et al.
**Axitinib**^**B**^	Fatigue, Diarrhea (45), Nausea (33), Anorexia (30), Stomatitis (25), Weight decrease (22)	Hypertension (12), Proteinuria, Fatigue (5), Headache, Weight decrease, Diarrhea (3)	Cohen et al.
**Dabrafenib +** **Trametinib**^**A**^	Fatigue (19–38), Nausea (25–31), Vomiting (13–25), Pyrexia (6–31), Constipation (6–25)	Anemia (6–13), Hypernatriemia (0–13), Fatigue (6), Diarrhea, Hypercalcemia, Hyperglicemia (0–6)	Subbiah et al. Iyer et al.
**Everolimus**^**B**^	Stomatitis/mucositis (46–69), Anorexia (26–42), Cough (0–60), Hyperglicemia (10–49), Anemia (0–57), Fatigue (0–51)	Mucositis (11–15), Hypertension (11), Diarrhea (10), Neutropenia (5), Anorexia (2–4)	Lim et al. Schneider et al.
**Spartalizumab**^**B**^	Diarrhea, Pruritus (12), Fatigue, Pyrexia (7), Anemia, Asthenia, Myalgia, Rash (5)	Anemia (5), Rash (2)	Capdevila et al.
**Fosbretabulin**^**A**^	Headache (51), Lymphopenia (34), Pain (other than tumor pain and headache) (32), Prolonged QTc (28), Tumor Pain (25)	Lymphopenia (11), Tumor Pain (4), Pain (other than tumor pain and headache)(1)	Mooney et al.^C^
**Sorafenib +** **Temsirolimus**^**B**^	NA	Hyperglicemia (19), Fatigue (14), Anemia (11), Alanine-aminotransferase increase, Oral mucositis (8)	Sherman et al.
**Fosbretabulin +** **CP**^**A**^	Alopecia (31), Fatigue, Hypertension (29), Nausea (24), Diarrhea (22), Anemia, Headache (20)	Neutropenia (43), Leukopenia (26), Anemia (16), Tumor pain (6	Sosa et al.
**Efatutazone +** **Paclitaxel**^**A**^	Edema (40), Anemia, Fatigue (33), Nausea, Weight increase, Dyspnea (27), Insomnia, Back pain, Abdominal pain, Cough (20)	Edema (13), Leukopenia, Neutrophil count decrased, Fatigue, Pneumonia, Hypovolemia (7)	Smallridge et al.^D^

Abbreviations: *AEs* Adverse Events; *CP*Paclitaxel followed by Carboplatin

Notes: ^A^AEs evaluated only in ATC patients

^B^AEs evaluated both in ATC and other histotype tumors

^C^Recurrence of AEs is expressed as the total number of events occurred during the study (all grade registered AEs were 477)

^D^AEs recurrence refers to all the enrolled patients, independently of the different therapeutic regimen

## Main text

### Conventional treatment

**Fig. 1:**
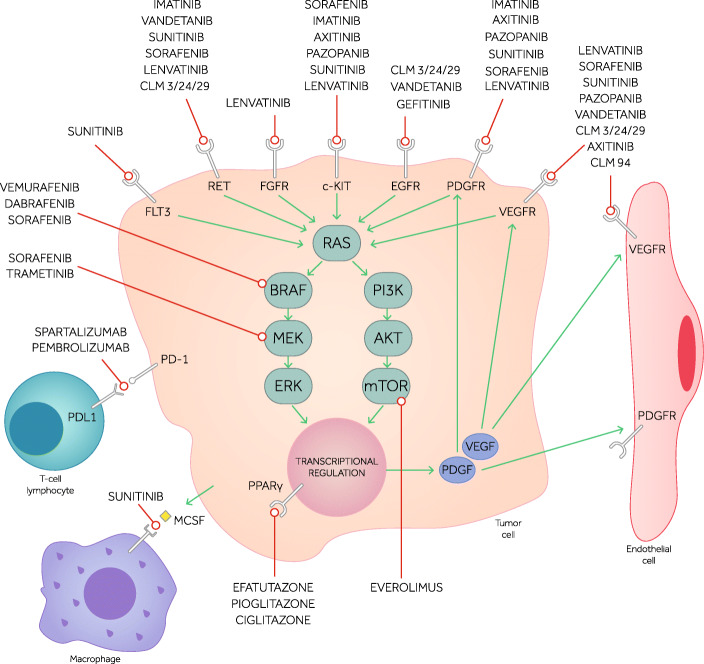
Molecular targets of targeted therapy in anaplastic thyroid cancer

Stage IV A tumors, which are localized to the thyroid gland, should be treated by thyroidectomy. A lobectomy may be performed, as long as a complete resection of the tumor is obtained [10]. However, the general accepted approach is total thyroidectomy because it has a higher probability of complete resection. The approach to stage IV B tumors depends on the feasibility to obtain a satisfactory resection (i.e. R0 resection, microscopic negative margins, or R1, macroscopic negative margins). ATC may invade vessels, nerves, muscles, esophagus, and trachea; therefore, preliminary assessment, by means of imaging studies (ultrasound, CT, MRI, PET) and possibly endoscopic exams, needs to be performed [6]. If the tumor is considered resectable, total thyroidectomy with prophylactic/therapeutic central and lateral neck lymph node compartments should be performed. An adjuvant radiotherapy with or without chemotherapy treatment is generally suggested in these cases. An aggressive treatment, whenever possible, has been associated to a better survival in various retrospective studies, even though the most useful results of an aggressive approach were reported in stage IV C tumors [11–16]. A total hyperfractionated dose of external beam radiotherapy should be higher than 40–45 Gy [11, 16, 17]; however, to date, the standard of care as regards radiotherapy is the use of the intensity-modulated radiation therapy (IMRT), to limit the damage to the surrounding normal structures [18, 19]. Radiotherapy is frequently associated to chemotherapy. Doxorubicin was historically used for its radiosensitizing effect [20]; more recently, other radiosensitizing agents have been used since they appear to be more effective than doxorubicin, in particular taxanes, such as paclitaxel and docetaxel, and platin, cisplatin and carboplatin, both alone and in combination [6]. If a stage IV B tumor is considered unresectable, neoadjuvant radiotherapy and/or chemotherapy should be considered [21]. Paclitaxel proved effective in stage IV B patients [22]. A multicenter, nonrandomized study demonstrated that Paclitaxel as neoadjuvant treatment was effective and tolerable, as 93% of patients could perform more than one cycle of treatment and none of them had to terminate it because of adverse effects (AEs). The median OS of all 56 ATC patients enrolled was 6.7 months. An ORR was obtained in 21.4% and a DCR in 73.8% of patients [23]. An open-label, single-center, prospective study enrolled seven patients (four of whom were not subjected to prior surgery) to evaluate feasibility and efficacy of docetaxel in controlling ATC. The AEs were manageable and the treatment proved effective, with an ORR of 14% and a DCR of 43%, suggesting a potential benefit from neoadjuvant use of docetaxel [24]. In patients with stage IV C ATC, the best initial treatment relies on the disease burden. Patients with a metastatic small disease burden may take advantage of IMRT and, when a rapid PD is recorded, a systemic treatment is needed; in patients with large metastatic disease burden the systemic treatment may be the first option [3]. Before starting with a systemic treatment, all ATC tumors should be evaluated by genetic analysis. Since the therapy may be tailored according to the genetic profile (Fig. 1), at least the BRAF mutation analysis should be performed. Whenever available, next-generation sequencing (NGS) analysis may be useful to have a full genetic characterization of the tumor and/or the metastases. When no mutation-directed therapy is possible and/or available, a multitarget kinase-inhibitor or immunotherapy may be used. Some of these drugs have been approved in some Countries (e.g. Dabrafenib and Trametinib treatment was approved in the United States, by the Food and Drug Administration (FDA), for ATC tumors with BRAF^V600E^ mutation; Lenvatinib was approved in Japan for ATC, independently from tumor mutational status); in other Countries, these drugs may be used either on a clinical trial (if available) or through a compassionate use program.

### Multitarget tyrosine-kinase inhibitors

#### Lenvatinib

Lenvatinib is an oral multitarget tyrosine-kinase inhibitor (TKI) that prevents signaling through several molecular pathways involved in tumoral angiogenesis: vascular endothelial growth factor (VEGF) receptor 1–3, fibroblast growth factor receptor (FGFR) 1–4, platelet-derived growth factor receptor (PDGFR) α, stem cell factor receptor (KIT), and rearranged during transfection (RET). It was approved by FDA for treating iodine-131 refractory differentiated thyroid carcinoma, as it demonstrated effective in improving progression-free survival (PFS) and response rate in these patients [25]. Antiproliferative activity of Lenvatinib against ATC cells has been confirmed both in vivo and in vitro [26]. In murine models of ATC tumors, the addition of anti-PD-1/PD-L1 to Lenvatinib is associated with consistent improvement in tumor reduction and in survival time [27].

A phase 2, single-arm, open-label study, in patients with advanced thyroid cancer, including ATC, was conducted to assess safety and efficacy of Lenvatinib administration. Seventeen patients with ATC were enrolled and treated with 24 mg of Lenvatinib per day; two patients did not receive any prior treatment. PFS was 7.4 months (95% CI: 1.7–12.9) and the median OS was 10.6 months (95% CI: 3.8–19.8); the ORR was 24%, the DCR 94%, and there was only one patient with progressive disease, suggesting a promising clinical activity in ATC for Lenvatinib. All patients experienced at least one AE (decreased appetite, hypertension, fatigue, nausea, and proteinuria being the most common). AEs were all effectively managed, in some circumstances requiring a dose reduction or a transient interruption, and Lenvatinib demonstrated a general manageable toxicity profile [28]. Lenvatinib was approved in Japan for the treatment of ATC in May 2015. In a study presented at the Japanese Society of Medical Oncology Annual Meeting, in 2019, the results of 124 ATC patients enrolled in all cases survey following this approval were reported: the ORR was 44.8%, the DCR 76.2%, and the survival rate at 1 year after Lenvatinib start was 18.5%. The results confirmed that Lenvatinib is effective and well tolerated in ATC patients in clinical practice [29].

A retrospective study evaluated 23 patients with IVC stage ATC treated with Lenvatinib. Ten patients previously underwent surgery, the remaining having not received any prior treatment. The ORR was 23.5% and the DCR was 58.8%. The median OS was 5.5 months and it was longer in the group of patients who underwent surgery compared to those without previous surgery: 8.7 vs. 4.3 months, although this difference was not statistically significant. AEs were observed in all patients and led to discontinuation in nine (39%) cases. [30]. Other groups of researchers experienced Lenvatinib potential efficacy as a salvage therapy for metastatic ATC [31–33], though clinical trials are required to ascertain its actual benefits. When administrated in surgery-naïve patients, Lenvatinib can more frequently bring to hypothyroidism [32] and fistulas [30], whose management is crucial to secure patients’ outcome.

#### Sorafenib

Sorafenib is an oral multitarget TKI that is active against VEGFRs (2 and 3), c-Kit, PDGFR, RET/ PTC, Raf kinases, and the Raf/Mek/Erk pathway (MAPK pathway). It has been approved for the treatment of unresectable hepatocellular carcinoma and advanced renal cell carcinoma; moreover, following the results of several phase II [34–37] and the phase III DECISION trial [38], Sorafenib has been approved for the treatment of locally recurrent or metastatic, progressive, differentiated thyroid carcinoma that is refractory to radioactive iodine treatment.

Sorafenib activity against ATC cells has been proved both in vitro and in vivo experiments. In the former, Sorafenib reduced proliferation and enhanced apoptosis in several ATC cell lines, regardless of BRAF mutation status [39, 40]; in the latter, Sorafenib proved effective in inhibiting the growth of orthotopic ATC xenografts in mice, thus improving their survival [39]. Preclinical studies suggested that several drugs may exert synergic effect with Sorafenib in reducing ATC cells growth, including metformin [41]. Moreover, a phase II clinical trial proposed the combination of Temsirolimus (an mTOR inhibitor) and Sorafenib as a possible alternative in RAI-refractory thyroid cancer, especially in patients who received no prior treatment. Two ATC patients were enrolled, one had a PD and one a PR [42]. A multicenter phase II study with Sorafenib 400 mg twice daily, conducted in the United States, enrolled 20 ATC patients with stage IV C disease. All received prior chemotherapy, and 18/20 were previously treated with surgery and radiation. The ORR and DCR were 10 and 35%, respectively; median PFS and OS were 1.9 months (CI 1.3–3.6) and 3.9 months (CI 2.2–7.1), respectively. [43]. Another multicenter phase II trial with Sorafenib 400 mg twice daily, carried out in Japan, enrolled 10 patients with ATC, the majority of whom previously treated by surgery, radiation, or systemic therapy. The ORR and DCR were 0 and 40%, while median PFS and OS were 2.8 (CI 0.7–5.6) and 5.0 months (CI 0.7–5.7) [44]. The most frequent AEs were palmar-plantar erythrodysesthesia, hypertension, weight loss, skin rash, fatigue, and electrolyte abnormalities, as expected, whereas hematological toxicities were uncommon [43, 44]. In conclusion, Sorafenib has a limited role in ATC, though it is possible that its combination with other targeted therapies may provide a more evident benefit.

#### Sunitinib

Sunitinib is an oral multitargeted TKI against VEGFRs (1 and 2), PDGFRs (α and β), c-KIT, FMS-like tyrosine kinase-3 (FLT3), glial cell-line derived neurotrophic factor receptor (RET) and the receptor of macrophage-colony stimulating factor (CSF1R). It has been approved for the treatment of advanced renal cell carcinoma and, after progression or intolerance to Imatinib, for the treatment of gastrointestinal stromal tumor (GIST) [45]. Several clinical trials have been conducted so far, showing a potential benefit of Sunitinib in the treatment of DTC and MTC [46, 47]. In vivo and in vitro studies reported contrasting evidences about a possible antiproliferative activity of Sunitinib on ATC cells [48, 49]. However, some molecules demonstrated a synergistic anti-neoplastic effect if associated to Sunitinib, such as SN-38 (an active metabolite of Irinotecan, whose role has been studied in vivo as well) [50] and SL327 (a MEK1/2 inhibitor) [51]. An open-label multicenter phase II trial has been conducted to assess efficacy and safety of Sunitinib in thyroid cancer treatment. Patients received Sunitinib at a starting dose of 50 mg per day for 4 weeks, followed by a 2-week rest. Only 4 out of 71 patients included had ATC and among them only 2 patients had a subsequent radiological assessment, with one recording a SD. Side effects were severe, suggesting alternative schedule/dosage: asthenia/fatigue, muco-cutaneous toxicities, and hand-foot syndrome were the most common AEs; life-threatening AEs and deaths consequent to the therapy were recorded [47]. Anecdotal use of Sunitinib for ATC treatment has been reported. A patient who developed ATC following total thyroidectomy and radioactive iodine for papillary thyroid cancer had a CR in the neck, whereas lung metastases remained stable. The patient died from a severe upper gastrointestinal bleeding after 5 months of Sunitinib treatment [52]. A woman operated on for ATC presented a gross residual disease treated with IMRT, along with chemotherapy (Docetaxel) and Sunitinib (37.5 mg daily for four weeks, followed by two weeks off). She had a CR and remained without evidence of disease more than 18 months after diagnosis [53]. In conclusion, too little data are available about Sunitinib use in ATC; probably this drug alone is not able to improve natural history of ATC.

#### Imatinib

Imatinib is a TKI inhibiting Bcr/Abl, PDGFR, c-Fms, c-Kit, and RET; it has been approved by FDA for the treatment of chronic myeloid leukemia, GIST, and dermatofibrosarcoma protuberans. Preclinical studies suggested potential benefit from Imatinib treatment in patients with ATCs, as it proved effective in inhibiting ATC cell lines both as a single therapy [54, 55], and as a part of combined therapy, along with Docetaxel (whose effect is enhanced by Imatinib through the NF-kB activity inhibition) [56] and Gefitinib [55].

A phase II clinical trial evaluated Imatinib (400 mg orally twice daily) efficacy in 11 patients with advanced ATC overexpressing PGDF receptors. Among the 8 patients evaluable for response, 25% had a PR (i.e. ORR of 25%) and 50% a SD (with a DCR of 75%); the estimate of 6-month PFS and OS were 27% and 46%, respectively. The most common AEs were anemia, fatigue, myalgia/arthralgia, and AST/ALT increase; among grade 3 toxicity, edema, fatigue, and hyponatremia occurred more frequently [57]. This drug may have a role in the treatment of ATC, though larger phase II and/or phase III trial are needed.

#### Pyrazolo[3,4-d]pyrimidine derivatives (CLM94, CLM3, CLM24, CLM29)

Pyrazolo [3,4-d]pyrimidine (PP) heterocyclic core has been proved to be a useful scaffold for obtaining effective TKI compounds. Different PP molecules have been shown to exert anti-tumor activity against thyroid cancer (PP1 and PP2 in particular); recently, new derivatives have been demonstrated to be active against different TC histotype, both in vitro and in vivo [58]. Among these, CLM3 is able to inhibit VEGFR-1, EGFR, and the RET tyrosine kinase. In vitro, it has significantly reduced proliferation, invasion, and migration of ATC cell lines, and increased apoptosis at the same time. In vivo, CLM3 (50 mg/kg per die) inhibited tumor growth and reduced microvessel density in xenograft models [59]. CLM24 and CLM29 have pharmacodynamic properties similar to CML3, as they inhibit RET, epidermal growth factor receptor (EGFR), and VEGF-R. CLM29, and at a slight level CLM24, were able to inhibit the proliferation of primary ATC cells, demonstrating an antineoplastic effect independently form the presence of BRAF^V600E^ mutation [60]. CLM94 is a cyclic amide with anti-VEGFR-2 and anti-angiogenic properties, which has been shown to be as active as CLM3 against MTC cells [59]. In experimental studies, CLM94 significantly inhibited migration, invasion, and tumor growth, and reduced microvessel density in ATC [61]. These compounds provided preclinical good results, but no clinical trials have been performed to date.

#### Other TKI drugs

*Vandetanib* is active against the EGFR family, VEGF receptors, RET, protein tyrosine kinase 6 (BRK), tyrosine kinase with immunoglobulin and EGF domains-2 (TIE2), members of the ephrin (EPH) receptor kinase family, and members of the Src family of tyrosine kinases. FDA approved Vandetanib for the treatment of unresectable, locally advanced, or metastatic MTCs, following the results of a phase III trial (ZETA study) [62]. Vandetanib proved effective in inhibiting primary ATC cells in vitro, by increasing apoptosis and reducing neoplastic cells’ migration and invasion capacity, and in vivo, reducing xenograft tumor growth, particularly through its antiangiogenic action [63, 64]. These evidences pave the way to future clinical evaluations, though neither clinical trials nor retrospective studies on Vandetanib efficacy in ATC-affected patients have been conducted so far.

*Axitinib* is a selective inhibitor of VEGFRs 1–3, and it has a weak activity against PDGFR-β, and c-KIT. In a phase II trial, Axitinib was used in 60 patients with advanced thyroid cancer, including two ATCs: one recorded a PR and the other a PD. [65]. The limited number of patients makes not possible a definitive conclusion concerning this drug.

*Pazopanib* is an inhibitor of VEGF receptors, PDGF, c-Kit, and other kinases, though less potent. Preclinical studies reported its efficacy in ATC both as a single agent and as part of a combined therapy with other chemotherapeutic agents [66–68]. However, a phase II clinical trial proved this compound ineffective in the treatment of ATC. Indeed, despite some consistent but transient responses, there were no stable tumor responses among the 15 patients enrolled [68].

### Anti-EGFR molecules

#### Gefitinib

Gefitinib is an EGFR inhibitor approved for the use in the advanced non-small cell lung cancer (NSCLC). ATC cells overexpress EGFR and experimental studies demonstrated a significant antitumor activity of Gefitinib in this tumor. In vitro studies reported that Gefitinib is able to inhibit proliferation and to increase apoptosis of ATC cell lines; in mice models, tumor growth was effectively impaired by the drug in a dose-dependent manner [69, 70]. A preclinical in vitro study has shown that radiation-induced inhibition of ATC cell line proliferation is enhanced when preceded by exposition to Gefitinib: this suggests that combination with Gefitinib could allow to use of lower doses of ionizing radiation, thus minimizing radiation toxicity [71]. Gefitinib has been tested in association with other drugs, too. A combination treatment with Imatinib provided good in vivo and in vitro results, since both molecules proved effective, but their activity was even greater when the two drugs were combined [55]. It has also been suggested that Gefitinib can enhance Doxorubicin toxicity in ATC cells, probably decreasing their ability to extrude Doxorubicin through the ABCG2 protein [72]. An open-label phase II trial has been conducted to assess the efficacy of Gefitinib (250 mg daily) in patients with advanced thyroid cancer. Among the 27 patients enrolled, 5 had ATC. There were no ORR among the patients evaluated, with SD for 12 month in 1 patient being the best response [73]. The activity of Gefitinib appeared to be modest, though further investigation may be needed to establish this TKI actual usefulness in thyroid cancer treatment.

### Anti-BRAF molecules

#### Vemurafenib

BRAF mutations (particularly, BRAF^V600E^) have been found in several human cancers. Vemurafenib, a selective BRAF inhibitor, has been approved by FDA for the treatment of patients with metastatic BRAF^V600E^-mutated melanoma, as it owns dose-dependent anti-proliferative and apoptotic effects in melanoma cells [74]. In 2017 it has been approved for the treatment of adult patients with Erdheim-Chester disease (ECD) with BRAF^V600E^ mutation [75]. Prospective and retrospective studies reported Vemurafenib efficacy in recurrent or metastatic PTCs, refractory to RAI and BRAF^V600E^-mutated. The most common AEs included rash, fatigue, weight loss, anorexia, dysgeusia, and alopecia [76, 77]. Preclinical studies showed that Vemurafenib downregulates angiogenic/cachectic and pro-inflammatory/immune response factors (IL-6, VEGFA, and VEGFC) that mediate microenvironment interactions between endothelial and ATC cells, thus inhibiting in vitro angiogenesis and lymphangiogenesis in ATC cells cultures [78]. Vemurafenib demonstrated also to be able to reduce ATC xenografts and metastasis growth in mice models [79]. A phase II basket study evaluated Vemurafenib efficacy in different BRAF^V600E^-mutated cancers: 122 patients were enrolled, 7 were affected by ATC. Among these, 1 patient had a CR and 1 had a PR, which was maintained for more than 12 months, none had SD [80]. Other anecdotal empirical uses of Vemurafenib treatment in ATC have been reported with positive results [81, 82].

#### Dabrafenib/Trametinib

Dabrafenib is a BRAF^V600E^ kinase signaling inhibitor, whereas Trametinib is a kinase inhibitor that blocks MEK, which is downstream of BRAF in MAPK pathway. Both these agents have been approved by FDA as monotherapy for patients with unresectable or metastatic melanoma harboring BRAF^V600E^ (and, for Trametinib, V600K) mutation. Within few months after inhibition of BRAF, several mechanisms allow the tumor to overcome this block and patients develop resistance to BRAF inhibitors. A combination treatment of a BRAF inhibitor and a MEK inhibitor has been tested in preclinical studies, and clinical trials with successful results in terms of both tumors response and toxic profile [83–85]. FDA approved this therapeutic association for the treatment of BRAF-mutated melanoma and NSCLC. In vitro studies evaluated Dabrafenib and Trametinib effect on different ATC cell lines. Dabrafenib inhibited cellular growth and seemed effective against tumors harboring mutations in both the MAPK/ERK and PI3K/AKT/mTOR pathways; Trametinib induced growth suppression independently by the mutational status of BRAF or NRAS [86]. The addition of a MEK inhibitor to a BRAF inhibitor (PLX4720) enhanced tumor regression and prolonged survival in ATC-bearing mice [87] Moreover, a combination therapy of BRAF^V600E^ inhibitor (PLX4720) and anti-PD-L1/PD-1 antibody reduced tumor volume and improved survival of an immunocompetent mouse model of orthotopic ATC [88].

A multicenter, open-label, nonrandomized, phase II trial evaluated the effectiveness and feasibility of Dabrafenib and Trametinib combination therapy, administered at doses of 150 mg twice daily and 2 mg once daily, respectively. Sixteen patients with ATC were enrolled: all had received prior radiation treatment and/or surgery and six had received prior chemotherapy; among them 15 had a confirmed BRAF^V600E^ mutation. Limiting the results to these 15 BRAF^V600E^ mutated ATC, the ORR was 73% (1 patient with CR and 10 with PR) and DCR 87%. Confirmed responses were durable since after 12 months of treatment PFS and OS were 79% and 80%, respectively, in the entire ATC cohort. The most common AEs were fatigue, pyrexia, and nausea; among SAE, acute kidney injury, rhabdomyolysis, and hyponatremia were recorded [89]. Retrospective studies confirmed this efficacy. A real-life monocentric experience was reported with the use of targeted therapy for ATC. Among the 16 patients included, six were treated with Dabrafenib and Trametinib, all of them having a BRAF^V600E^ positive ATC. In these patients, ORR was 50% and DCR 83.3% (3 PR and 2 SD), and PFS and OS were 5.2 months (CI 3.7-NR) and 9.3 months (CI 5.7-NR), respectively. AEs were tolerable, with nausea, fatigue, hand–foot skin reaction, hyponatremia, anemia, and weight loss being the most frequent [33]. Dabrafenib plus Trametinib combination has been evaluated as neoadjuvant regimen in patients with initially unresectable BRAF^V600E^-mutated ATC. In a retrospective cohort of six patients, the combination treatment proved effective to achieve a surgical complete resection in advanced inoperable ATC disease. In this study, three patients also received Pembrolizumab (before or after resection) and surgery was followed by adjuvant chemoradiation. Complete resection was achieved in all patients; OS at six months and one year was 100% and 83%, respectively. Two patients died of distant metastases, the remaining had no evidence of disease at the last follow-up [90]. In conclusion, Dabrafenib plus Trametinib proved to be clinically active and well tolerated and it may have a role as neoadjuvant treatment in BRAF^V600E^ mutated ATC patients. After these encouraging results, FDA approved the combination of Dabrafenib and Trametinib for BRAF^V600E^-mutated ATC.

### Anti-mTOR molecules

#### Everolimus

Constitutive activation of the phosphatidylinositol-3-kinase (PI3K)/AKT/mTOR pathway has been reported in thyroid cancer pathogenesis. Everolimus is a Sirolimus-derived mTOR inhibitor, whose activity against several thyroid cancer cell lines has been confirmed both in vitro and in vivo [91]. In experimental studies, Everolimus provided growth inhibition in ATC cell lines harboring a PI3K mutation, which made those cells Gefitinib-resistant, thus suggesting a possible correlation between its efficacy and PI3K/Akt/mTOR signaling pathway alteration [92]. A multicenter, phase II trial evaluated efficacy and safety of Everolimus, administered at a 10-mg daily dose, in patients with advanced thyroid carcinoma of any histology. Among the 38 patients enrolled, 6 had ATC. None of the patients with ATC had a CR or PR, but one had marked tumor reduction and PFS was 10 weeks (CI 4.8–16) [93]. A phase II trial, conducted in The Netherlands, enrolled 7 patients with advanced ATC who received Everolimus 10 mg once daily. All these patients had a PD [94]. Another nonrandomized, single-arm, phase II trial enrolled 50 patients with advanced thyroid cancer of any histology, among them 7 had ATC. In ATC patients, ORR was 14% (one had PR) and DCR 42% (with 2 patients having a SD). Median PFS was 2.2 months (CI: <1-17.9) and median OS 4.6 months (CI < 1-29.9). A tumor mutational analysis was performed. The patient with PR had a near-complete response and it was found to have a nonsense mutation of the tumor suppressor gene TSC2 (tuberous sclerosis complex 2). Moreover, in the subgroup PDTC/ATC who were sequenced, median PFS was 2.8 months, but it was definitely longer if a PI3K/mTOR/Akt mutation was detected (median PFS of 15.2 months) [95]. A retrospective study on five ATC patients treated by Everolimus reported a PR in one patient who had PI3KCA mutations (besides BRAF) [96] These results need confirmation in larger studies but suggest that ATC may benefit most from Everolimus when a mutation involving the PI3K/mTOR pathway is present. Everolimus is well tolerated and the most frequent AEs reported are mucositis, anorexia, acneiform rash, anemia, thrombocytopenia, and transaminase elevation [93–95].

### PPARγ ligand molecules

PPARγ are nuclear hormone receptors and their role in tumorigenesis is still controversial. PPARγ has been shown to be abundantly expressed in ATC cells, whereas it is virtually absent in cells from more differentiated thyroid cancers. PPARγ knock-down in an ATC cell line determines in vitro inhibition of ATC cell growth, as well as in vivo reduction of growth in xenograft tumors [97]. On the other hand, reduced PPARγ protein has been shown to determine the activation of cyclin D1 and repression of critical genes involved in apoptosis, thus promoting thyroid carcinogenesis [98]. Various preclinical studies have highlighted a potential role for Thiazolidinediones (TZDs), synthetic agonists of PPARγ, for the treatment of cancer. Pioglitazone [99], Ciglitazone [100], and Rosiglitazone [99, 100] have been shown to exert antiproliferative/pro-apoptotic effect in ATC cells. Efatutazone is a selective and potent agonist of PPAR ligands and its efficacy in ATC has been tested in a phase I study, where the drug was administered at different doses in combination with Paclitaxel. This combination was considered safe and well-tolerated [101]. Fifteen patients were enrolled and treated with 0.15 mg (*n* = 7), 0.3 mg (*n* = 6), and 0.5 mg (*n* = 2) twice daily. ORR and DCR were 7% and 53%. Median PFS was 48 and 68 days in the 0.15 and the 0.3 mg, respectively.

### Vascular disruptor molecules

Combretastatin A4 (CA4), also known as Fosbretabulin, is a natural product isolated from the tree *Combretum caffrum*. CA4 has an antimitotic action that can cause vascular shutdown and cell death in tumors; it has been demonstrated to have cytotoxic and anti-proliferative activity in a variety of human cancer cells. Preclinical studies reported cytotoxicity of CA4-phospate comparable to Paclitaxel against ATC cell lines and xenografts tumors in nude mice, probably as a consequence of both anti-neoplastic effects and destruction of tumor vasculature [102]. In vivo studies demonstrated that triple-drug therapy (CA4P, Paclitaxel, and Manumycin as well as CA4P, Paclitaxel, and Carboplatin) exerts excellent antineoplastic activity against ATC xenografts [103]. A phase II trial assessed single-agent Fosbretabulin efficacy in 26 patients with advanced ATC, administered at a dose of 45 mg/m^2^ as a 10-minute intravenous infusion on days 1, 8, and 15 of a 28-days cycle. No patient obtained an objective response, and 27% had a SD; median OS was 4.7 months (CI 2.5–6.4) and it was 12.3 months (CI 4.4–37.9) among patients with SD. Therapy was generally well tolerated with the most common AEs being nausea, vomiting, and headache; 15% of patients had a QTc interval prolongation [104]. The FACT trial, an open-label, randomized, multicenter study evaluated safety and efficacy of carboplatin/paclitaxel (CP) with or without Fosbretabulin in ATC. Among the 80 patients enrolled, 55 were randomized in the CP/Fosbretabulin arm, the remaining were enrolled in the control group (CP arm). Patients received Fosbretabulin 60 mg/m^2^ on days 1, 8 and 15 and Paclitaxel and Carboplatin on day 2 of each 21-day cycle, while those in the CP arm received Paclitaxel 200 mg/m^2^ followed by Carboplatin on day 1 every 3 weeks. PR and SD were recorded in 20 vs. 16% and 40 vs. 44% in the arms CP/Fosbretabulin vs. CP, respectively. Median OS was 5.2 months (CI 3.1-9.0) for the CP/Fosbretabulin arm and 4.0 months (CI 2.8–6.2) for the CP arm (p = 0.22); Median PFS was similar for both treatments: 3.3 (CI 2.3–5.6) vs. 3.1 months (CI 2.7–5.4). The addition of Fosbretabulin to CP did not provide a significant improvement in terms of OS, though the drug may have a clinical activity in these patients with a good safety profile [105].

### Checkpoint inhibitor drugs targeting PD-1 or PD-L1

Programmed cell-death 1 (PD-1) is a glycoprotein normally expressed by macrophages and T-cells. The binding of PD-1 to its ligands (PD-L1 or PD-L2) inhibits cytotoxic T-cell immune response and leads to an immune escape of the cells that express these ligands. These may be constitutively present on tumor cells, in particular it was found in up to 65–90% of ATC cells [106–108]. Preclinical studies demonstrated that PD-1/PD-L1 blockade was effective in reducing tumor growth [108] and, when anti-PD-1/PD-L1 antibody was administered in combination with a BRAF-inhibitor, it reduced tumor volume and prolonged survival in murine models [88]. Furthermore, in vivo study showed that inhibition of PD-1/PD-L1 pathway enhances Lenvatinib anti-tumor activity, through modification of ATC microenvironment [27]. A phase II clinical trial evaluated efficacy and tolerability of Spartalizumab (a humanized monoclonal antibody that binds PD-1 and blocks interaction with PD- L1) in 42 ATC patients. Spartalizumab was administered at a dosage of 400 mg intravenous every 4 weeks. ORR and DCR were 19% and 31%; median PFS and OS were 1.7 and 5.9 months, respectively. In the subgroup of patients with PD-L1 expression, ORR was 29%, and it was even higher when only patients with strong intensity PD-L1 expression were analyzed. These results were independent from the BRAF mutational status. AEs occurred in almost all patients, the most frequent being diarrhea, pruritus, fatigue, and pyrexia [109]. In conclusion, Spartalizumab proved to be a valid therapeutic option in patients with PD-L1-positive advanced ATC.

Pembrolizumab, a selective anti-PD-1 monoclonal antibody, was tested in advanced differentiated thyroid cancer [110], but in ATC patients, in combination with chemoradiation, it reported discouraging results [111]. In a retrospective study, Pembrolizumab was evaluated in association with other TKIs. Twelve ATC patients received Pembrolizumab in addition to Lenvatinib (*n* = 5), Dabrafenib plus Trametinib (*n* = 6), or Trametinib (*n* = 1), at the time of progression on kinase inhibitors. PR and SD in patients treated with Pembro + Lenvatinib were 60 and 20%, and in those receiving Pembro + Dabrafenib + Trametinib 17 and 67%, respectively [112]. Pembrolizumab appears to be an effective salvage therapy in addition to other TKIs. Several prospective studies are ongoing with immune checkpoint inhibitors in advanced ATC. These include the use of Pembrolizumab (NCT02688608); Pembrolizumab with Lenvatinib (NCT04171622); Atezolizumab with Vemurafenib (in the cohort of patients with BRAF mutations), Cobimetinib (in the cohort of patients with RAS or NF1-2 mutations), or Bevacizumab (if no BRAF and RAS mutations) (NCT03181100); Nivolumab with Ipilimumab (NCT03246958); EBRT with Durvalumab and Tremelimumab (NCT03122496) and EBRT with Ipilimumab (NCT02239900).

## Conclusions

ATC is generally managed with a combination of surgery, chemotherapy, and radiotherapy; however, its prognosis is still dire. Several treatments have been tested in the last two decades; these include inhibition of single or multi-kinase receptors and pathways, vascular disruption, and immunotherapy. The approach to ATC is now moving towards a personalized medicine, tailored to the clinical characteristics and genetic profile of the patients. Consistently, the genetic evaluation of the primary and/or metastatic tissue is becoming more and more crucial. BRAF mutation analysis is mandatory, since, if a BRAF^V600E^ mutation is detected, treatment with Dabrafenib and Trametinib is preferred. However, NGS should be the gold standard, since finding of other mutations can make a treatment preferable to another: for example, Everolimus if a mutation involving the PI3K/mTOR pathway is present, Imatinib in case of overexpression of PGDF receptors, or Spartalizumab in PD-L1 positive tumors. In patients with no mutations detected or unavailability of other molecules, Lenvatinib is the treatment of choice, since it provides the best results and is probably more widely available. Particular attention deserves the combination treatments with two or more molecules, in addition to conventional chemotherapy and radiotherapy. Several pathways may be inhibited, improving the drug response and reducing toxicities (since doses can be reduced). Several clinical trials are ongoing with this aim, and it is likely that the near future will provide more data in this particular setting. However, the improvement in the survival of these patients seems still to be a very difficult task, since to date even the molecules with the best results reported a not significantly durable disease control, apart from some anecdotal cases.

## Data Availability

Data sharing is not applicable to this article as no datasets were generated or analyzed during the current study.

## Abbreviations

AE: Adverse Event
AJCC: American Joint Committee on Cancer
ALT: Alanine Transaminase
AST: Aspartate Transaminase
ATC: Anaplastic Thyroid Cancer
BRK: Breast tumor Kinase
CA4: Combretastatin A4
CA4P: CA4-phospate
c-Fms: Colony-stimulating factor-1 receptor
CP: Paclitaxel followed by Carboplatin
CR: Complete Response
CSF1R: Colony Stimulating Factor 1 Receptor
CT: Computed tomography
CTX: Chemotherapy (including both traditional and novel drugs)
DCR: Disease Control Rate
DTC: Differentiated Thyroid Cancer
EGFR: Epidermal Growth Factor Receptor
ECD: Erdheim-Chester disease
EKR: Extracellular signal-regulated kinases
EPH: Ephrine
FDA: Food and Drug Administration
FLT3: Fms-Like Tyrosine-kinase 3
GIST: Gastrointestinal stromal tumor
IMRT: Intensity-modulated radiotherapy
MAPK: Mitogen-activated protein kinase
MEK: Mitogen/Extracellular signal-regulated Kinase
MRI: Magnetic resonance imaging
MTC: Medullary Thyroid Cancer
mTKI: Multiple Tirosine-Kinase Inhibitor
mTOR: Mammalian Target Of Rapamycin
NF-κB: Nuclear factor kappa-light-chain-enhancer of activated B cells
NGS: Next Generation Sequencing
NSCLC: Non-small-cell lung carcinoma
ORR: Overall Response Rate
OS: Overall Survival
PP: Pyrazolo [3,4-d]pyrimidine
PPARγ: Peroxisome Proliferator-Activated Receptor Gamma
PD: Progressive Disease
PD-1: Programmed cell Death protein 1
PD-L1/2: Programmed death-ligand 1/2
PDGF: Platelet-Derived Growth Factor
PDGFR: Platelet-Derived Growth Factor Receptor
PDTC: Poorly Differentiated Thyroid Cancer
PET: Positron Emission Tomography
PFS: Progression Free Survival
PI3K/PI3KCA: Phosphatidylinositol 3-Kinase
PR: Partial Response
PTC: Papillary Thyroid Cancer
RAF: Rapidly Accelerated Fibrosarcoma
RAI: Radioactive iodine
RECIST: Response evaluation criteria in solid tumors
RET: REarranged during Transfection
SD: Stable Disease
TIE2: Tyrosine kinase with immunoglobulin-like and EGF-like domains 2
TKI: Tirosine-Kinase Inhibitor
TSC2: Tuberous Sclerosis Complex 2
TZDs: Thiazolidinediones
VEGF: Vascular Endothelial Growth Factor
VEGFR: Vascular Endothelial Growth Factor Receptor
XRT: Radiotherapy
